# Pharmacological Inhibition of JNK Signalling Exerts Anti-Neoplastic Effects on SH-SY5Y Human Neuroblastoma Cells

**DOI:** 10.3390/ijms262411894

**Published:** 2025-12-10

**Authors:** Zuzanna Granek, Natalia Siwecka, Kamil Saramowicz, Grzegorz Galita, Michał Golberg, Ireneusz Majsterek, Wioletta Rozpędek-Kamińska

**Affiliations:** 1 Department of Clinical Chemistry and Biochemistry, Medical University of Lodz, Mazowiecka 5, 92-215 Lodz, Poland; zuzanna.granek@stud.umed.lodz.pl (Z.G.); natalia.siwecka@stud.umed.lodz.pl (N.S.); kamil.saramowicz@stud.umed.lodz.pl (K.S.); grzegorz.galita@umed.lodz.pl (G.G.); ireneusz.majsterek@umed.lodz.pl (I.M.); 2 Department of Histology and Embryology, Medical University of Lodz, 92-215 Lodz, Poland; michal.golberg@umed.lodz.pl; 3 Department of Child Psychiatry, Children’s Clinical Hospital, Medical University of Warsaw, 02-091 Warsaw, Poland

**Keywords:** neuroblastoma, c-Jun N-terminal kinase, JNK inhibitor, small-molecule inhibitor, anticancer drug, cytotoxicity, apoptosis, mitochondrial bioenergetics

## Abstract

Neuroblastoma (NB) is the most prevalent paediatric extracranial solid tumour, which remains a major therapeutic challenge, especially in cases of recurrent and disseminated disease. c-Jun N-terminal kinases (JNKs) are increasingly evidenced to play a key role in NB tumourigenesis and progression through apoptosis regulation, making selective JNK inhibitors promising candidates for use in targeted anticancer drugs in NB. Our study comprehensively investigated the acute antineoplastic potential of the selective JNK inhibitor AS601245 (JNK inhibitor V) on the human *MYCN*-non-amplified neuroblastoma cell line, SH-SY5Y, with particular focus on its effects on NB cell viability, proliferation, migration, apoptosis, gene and protein expression, and mitochondrial metabolism. JNK V selectively impaired NB cell survival and function, without exerting cytotoxicity toward normal human Schwann cells (HSC) and fibroblasts (BJ). Our findings highlighted a dose-dependent inhibition of proliferation (XTT assay), colony formation (clonogenic assay), and migration (wound healing assay), accompanied by increased caspase-3 activity (caspase-3 assay), pro-apoptotic genes (qRT-PCR) and protein (Western blotting) expression, and significant disruption of both oxidative phosphorylation and glycolysis (Agilent Seahorse XF Assay). These results provide new insights into the therapeutic potential of JNK inhibition as a targeted strategy for NB.

## 1. Introduction

Neuroblastoma (NB) is the most frequently occurring paediatric extracranial solid tumour, constituting up to 15% of all cancer deaths in children [1]. NB is an embryonal neuroendocrine neoplasm of the sympathetic nervous system, originating from neural crest progenitor cells, which undergo maladaptive differentiation because of genomic and epigenetic defects [2]. *MYCN* oncogene amplification is the first independent prognostic factor indicating poor clinical outcomes of NB patients. However, it occurs in only approximately 18% of neuroblastoma patients, while nearly 80% present with single-copy *MYCN*—a subgroup that includes low- and intermediate-risk NB and remains comparatively less explored [3,4]. Given this clinical distribution, our primary aim was to directly address this research gap and investigate the effects of the JNK inhibitor in the context of the more prevalent *MYCN*-non-amplified disease, relevant to the broad majority of NB patients [5].

Treatment of NB remains challenging because of its high genetic, immunological, and clinical heterogeneity, suboptimal response to initial therapy, high risk of relapse, and growing chemoresistance of tumour cells [6,7,8]. One promising therapeutic strategy against NB is the pharmacological inhibition of c-Jun N-terminal kinases (JNKs), as they converge on many pathways and are considered the main regulators of apoptosis in tumours [9,10,11,12,13].

JNKs constitute a group of Mitogen-Activated Protein Kinases (MAPKs) that regulate cancer cell survival and apoptosis [14,15]. *MAPK8*, *MAPK9*, and *MAPK10* genes encode the three JNK isoforms, respectively, JNK1, JNK2, and JNK3, which were reported to have multifaceted roles [16,17,18]. The experimental studies on NB indicated that the JNK1 isoform performs a pro-proliferative role, while JNK2 and JNK3 are assumed to play a pro-apoptotic role [19,20]. JNKs control the expression of cell cycle-, apoptosis-, mitochondria-, and ER stress-related genes, playing a crucial role in executing cell death in response to various apoptotic stimuli, both transcriptionally and through transcription-independent mechanisms [21,22]. JNKs undergo mitotic translocation, which results in the discharge of cytochrome c (Cyt c) and the Second Mitochondria-derived Activator of Caspase (SMAC), both of which directly induce apoptosis [21]. However, the precise mechanism of the JNK pathway’s role has not been fully elucidated in terms of NB.

Although JNK is known as a well-evidenced apoptosis kinase, under specific stress conditions in the tumours, in which JNK is overexpressed, it may evoke a paradoxical effect and contribute to cancer cell survival and chemotherapy resistance and the inhibition of cancer cell apoptosis [12,22]. Emerging evidence indicated that transiently activated JNK promotes cancer cell survival by modulating JNK-Bcl-2/Bcl-xL-Bax/Bak- as well as Cyt-C/caspase-3-dependent pathways [19,23,24]. Studies have demonstrated that both genetic inactivation of JNK signalling and specific pharmacological JNK inhibition can suppress tumorigenesis [25,26]. Hence, multiple JNK inhibitors have recently been widely investigated as a potential anticancer treatment, alone and in combination with chemotherapy [27,28]. Recent data indicated the enormous potential of JNK inhibitors in NB treatment, as they have been reported to induce NB cell death via direct activation of p53-, JNK-, Bcl-2-, and caspase-dependent pathways [29,30].

Based on available evidence, this is the first comprehensive study that investigates the antineoplastic effect of JNK inhibition in the NB in vitro model, a SH-SY5Y human neuroblastoma cell line, using the pharmacological inhibition of JNK signalling by JNK inhibitor AS601245 (JNK inhibitor V, JNK V). This selective small-molecule inhibitor exerts the strongest effect on the least-studied isoform, JNK3, which is hypothesised as a target in NB due to its expression in neuronal tissue and potential molecular involvement in apoptosis and oxidative stress response [19,31]. The study provides novel preclinical evidence supporting the further development of JNK inhibitors in the treatment of NB.

## 2. Results

### 2.1. Evaluation of the Cellular Toxicity of the JNK Inhibition on SH-SY5Y, HSC, and BJ Cells

**Figure 1:**
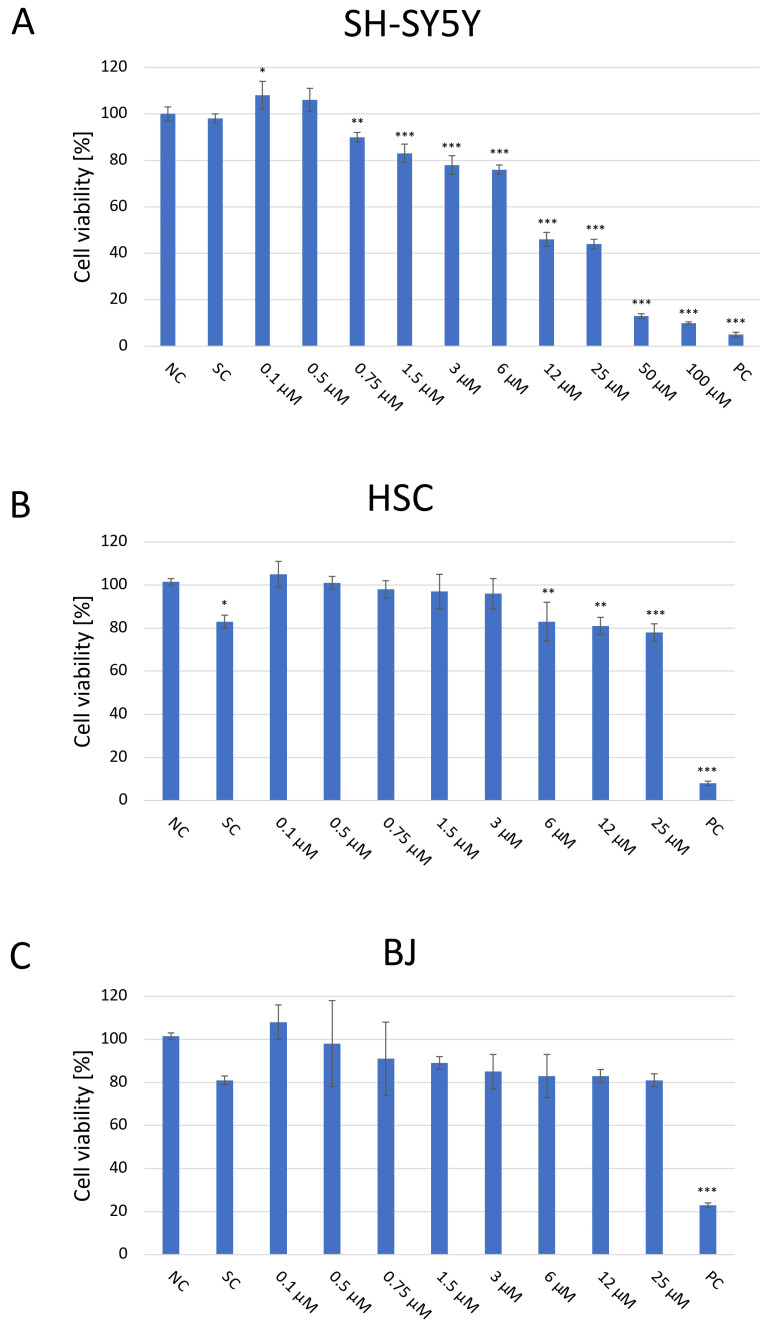
Analysis of the cytotoxicity of the JNK V inhibitor at 0.1–100 µM after incubation for 24 h toward SH-SY5Y (**A**), HSC (**B**), and BJ (**C**) cells performed by the XTT assay. NC—negative control (untreated cells); SC—solvent control (cells treated with 0.01% DMSO); PC—positive control (cells treated with 20% DMSO). All experiments were run in triplicate. Statistics: One-way ANOVA with Bonferroni post hoc test (mean ± SD). * *p* < 0.05, ** *p* < 0.01, *** *p* < 0.001 vs. negative control.

The cytotoxic effect of JNK V on three cell lines was evaluated using a colorimetric XTT assay (Figure 1). The cells were then treated and incubated with a particular compound for 24 h to assess the acute short-term effects of the investigated compound. The cytotoxicity experiment revealed significant inhibition of the proliferation of SH-SY5Y cells following the 24 h incubation with JNK V at concentrations ≥ 0.75 µM compared with the negative control. The cytotoxic effect was significant, and SH-SY5Y cells’ viability was reduced by almost 60% at 12 µM JNK V. In our experiment on SH-SY5Y cells, the IC50 of JNK V was 9 μM after 24 h of incubation. Therefore, the particular JNK V concentration ranges for all further experiments were determined based on XTT assay results, using the methods’ dedicated specifications. Low concentrations of JNK V (0.1–3 μM), reducing cell viability up to 30% after 24 h, were chosen for migration experiments, moderate concentrations for clonogenic assay (0.19–25 μM) and mRNA expression analysis (1–25 μM), and higher concentrations for caspase-3 activity assessment (0.75–100 μM), protein expression, and mitochondrial function analysis (1–100 μM). A solvent for JNK V, 0.01% DMSO, did not induce significant toxicity in SH-SY5Y cells after 24 h of incubation compared to the negative control. Furthermore, on normal cell lines, HSC and BJ, the JNK V did not demonstrate a cytotoxic effect. Although in HSC, the decrease in cell viability at JNK V ≥ 6 μM was statistically significant compared to the negative control, the viability of HSCs at all tested concentrations was maintained ≥78%, which is above the ISO 10993-5 cytotoxicity threshold [32]. HSC is a primary glial cell line characterised by high sensitivity to treatment-induced stress, which may explain the observed but non-cytotoxic reduction in viability. Moreover, the viability of BJ fibroblasts, which are much more resilient cells, was not significantly affected by JNK V at all tested concentrations, nor at 0.01% DMSO. Therefore, the XTT assay revealed the selective cytotoxicity of JNK V toward NB cells.

### 2.2. Evaluation of the Morphology of SH-SY5Y Cells After JNK Inhibition

**Figure 2:**
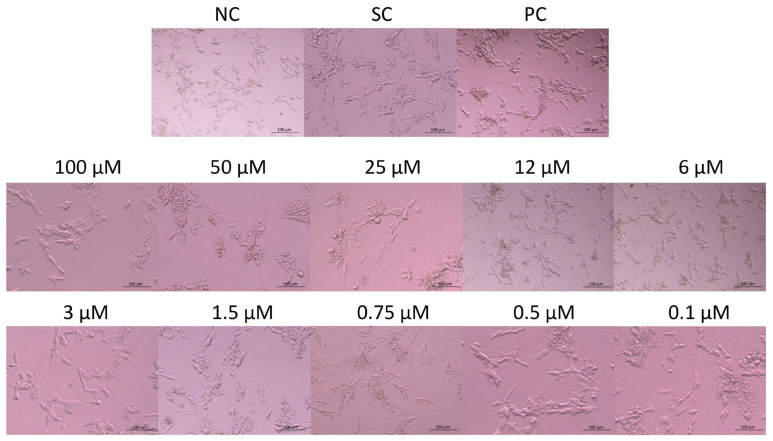
Representative images of SH-SY5Y cells’ morphology after 24 h of incubation with JNK V inhibitor at 0.1–100 µM. NC—negative control (untreated cells); SC—solvent control (cells treated with 0.01% DMSO); PC—positive control (cells treated with 20% DMSO).

The morphology of the cells exposed to JNK V, 0.01% DMSO, or 20% DMSO was assessed after 24 h of incubation using an inverted microscope (Nikon, Tokyo, Japan) (Figure 2). Untreated SH-SY5Y cells (negative control) exhibited a characteristic neuronal-like morphology with elongated neurites, an adherent and polygonal cell shape, and a homogenous cytoplasm. Cells treated with JNK V demonstrated concentration-dependent morphological alterations, from moderate shrinkage, the loosening of cell–cell contact, and neurite retraction at <3μM JNK V concentrations to increased cell detachment, loss of neurites, and nuclear condensation at 3–12 μM JNK V to loosening of the capacity to attach to the culture vessels and widespread cell lysis at JNK V > 12 μM. The results demonstrated a markedly visible impairment of the morphology of SH-SY5Y cells after treatment with JNK V at a wide concentration range, evidencing the cytotoxic effect of the compound on NB cells.

### 2.3. Evaluation of the Colony-Forming Abilities of SH-SY5Y Cells After JNK Inhibition

**Figure 3:**
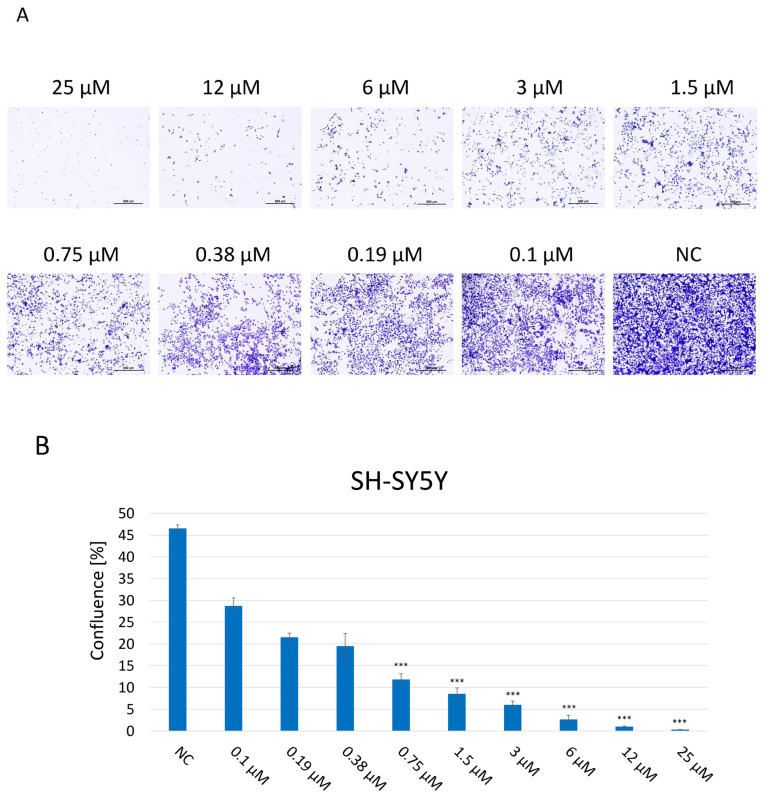
(**A**) Representative images of proliferation and colony-forming abilities of SH-SY5Y cells incubated for 2 weeks with JNK V inhibitor at 0.1–25 µM by clonogenic assay; NC—negative control (untreated cells). Scale bar: 500 µm. (**B**) Evaluation of the confluence of new colonies formed; NC—negative control (untreated cells). All experiments were run in triplicate. Statistics: One-way ANOVA with Dunnett’s post hoc test (mean ± SD); *** *p* < 0.001 vs. negative control.

A clonogenic assay was performed in order to assess the proliferation and colony-forming abilities of SH-SY5Y cells, indicating their long-term reproductive capacity and overall viability after 2 weeks of incubation with JNK V at 0.1–25 μM (Figure 3). Untreated cells maintained undisturbed proliferation, reaching 100% confluence, and properly formed colonies. The assay results showed that JNK inhibition significantly impaired the proliferation and colony-forming abilities of SH-SY5Y cells in a dose-dependent manner, compared to untreated cells, which reached 47% confluence at the final time point. Cells exposed to JNK V, even at the very low concentration of 0.1 μM, reached 29% confluence, while the confluence of cells treated with JNK V concentrations ≥1.5 μM was <10% at the final time point. Treatment with the inhibitor at a high concentration of 25 µM completely suppressed the cells’ proliferation, calculated at 0.3% confluence, and led to no colony formation, indicating the strong cytotoxicity of the compound. The results showed the impairment of long-term proliferation and survival of SH-SY5Y cells by pharmacological JNK inhibition, showing its cytotoxic and antineoplastic potential against cancerous NB cells.

### 2.4. Evaluation of the Migratory Abilities of SH-SY5Y Cells After JNK Inhibition

**Figure 4:**
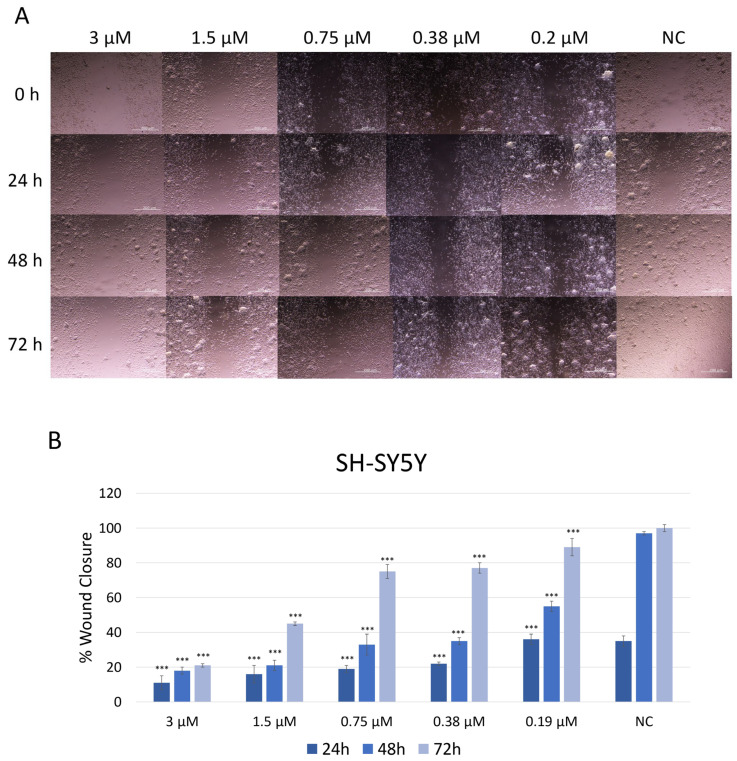
(**A**) Representative images of SH-SY5Y cells’ migration by wound healing assay after treatment with JNK V inhibitor at 0.19 μM, 0.38 μM, 0.75 μM, 1.5 μM, and 3 μM concentrations. Photos were taken at particular timepoints: 0 h, 24 h, 48 h, and 72 h. (**B**) Evaluation of the percentage of wound closure. NC—negative control (untreated cells). All experiments were run in triplicate. Statistics: One-way ANOVA with Bonferroni post hoc test (mean ± SD); *** *p* < 0.001 vs. negative control.

The effect of JNK V on the migration ability of SH-SY5Y cells was assessed using a wound healing (scratch) assay (Figure 4). Cells treated with JNK V at low and moderate concentrations (0.19 µM to 3 µM) closed the mechanically made scratch relatively more slowly compared to untreated cells. Phase-contrast microscopy revealed that inhibitor-treated cells retained their morphology but exhibited reduced motility, as indicated by fewer cells at the wound edges and a wider residual gap. In the control group (untreated cells), the wound area gradually decreased over time, with 38% wound closure after 24 h and almost complete wound closure (97%) after 48 h, indicating active cell migration. The treatment with JNK V at all tested concentrations resulted in significantly impaired cell migration across all time points, compared to negative control cells. At all tested JNK V concentrations, cells reached <60% wound closure after 48 h. SH-SY5Y cells reached only 21% of wound closure after 72 h of treatment with JNK V at 3 µM and less than 50% of wound closure after 72 h of incubation with 1.5 µM JNK V, indicating a strong anti-migratory effect of the inhibitor. These results suggest that JNK V effectively suppresses SH-SY5Y cells’ invasion abilities in a concentration-dependent manner, indicating the anti-migratory and further anti-metastatic potential of the pharmacological JNK inhibition in NB cells.

### 2.5. Evaluation of Caspase-3 Level in SH-SY5Y Cells After JNK Inhibition

**Figure 5:**
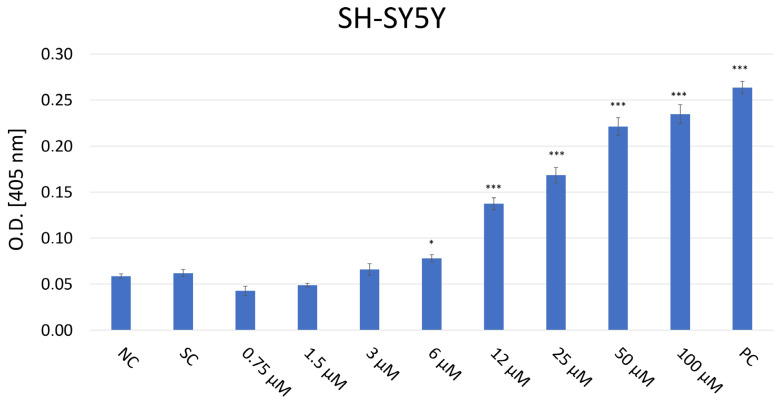
Analysis of caspase-3 level in SH-SY5Y cells after 24 h incubation with JNK V inhibitor at 0.75–100 µM by Caspase-3 colorimetric assay. The absorbance of the chromogenic reaction product values at 405 nm corresponds to the caspase-3 enzymatic activity. NC—negative control (untreated cells); SC—solvent control (cells treated with 0.01% DMSO); PC—positive control (cells treated with 10 µM staurosporine). All experiments were run in triplicate. Statistics: Kruskal–Wallis one-way analysis of variance on ranks with Bonferroni *t*-test (mean ± SD); *** *p* < 0.001 and * *p* < 0.05 vs. negative control.

The caspase-3 colorimetric activity assay was conducted to evaluate the activity of the caspase-3 enzyme as an indicator of apoptosis in SH-SY5Y cells treated with JNK V inhibitor at a concentration range of 0.75–100 µM (Figure 5). Although JNK is typically known to activate caspase-3, its inhibition may paradoxically increase caspase-3 levels in particular cancer and neuronal cells under chronic stress conditions by disinhibiting other pro-apoptotic signals such as p38 or intrinsic mitochondrial pathways, triggering the activation of caspase-9 as well as downstream caspase-3 [33]. The absorbance of the chromogenic reaction product, p-nitroaniline (pNA), at 405 nm corresponds to the caspase-3 enzymatic activity in cells. The obtained results demonstrated a significant elevation in the caspase-3 activity level in cells treated with JNK V inhibitor ≥ 6 µM after 24 h of treatment, compared to the baseline level of caspase-3 in untreated cells. The absorbance values in untreated cells showed minimal caspase activity (O.D. = 0.055), whereas treatment with 100 μM of the inhibitor resulted in a 4.3-fold increase in enzyme activity (O.D. = 0.240). The result evidenced that pharmacological JNK inhibition promotes caspase-dependent apoptosis in NB cells.

### 2.6. mRNA Expression Analysis of the Apoptosis-Related Genes in SH-SY5Y Cells After Pharmacological JNK Inhibition

**Figure 6:**
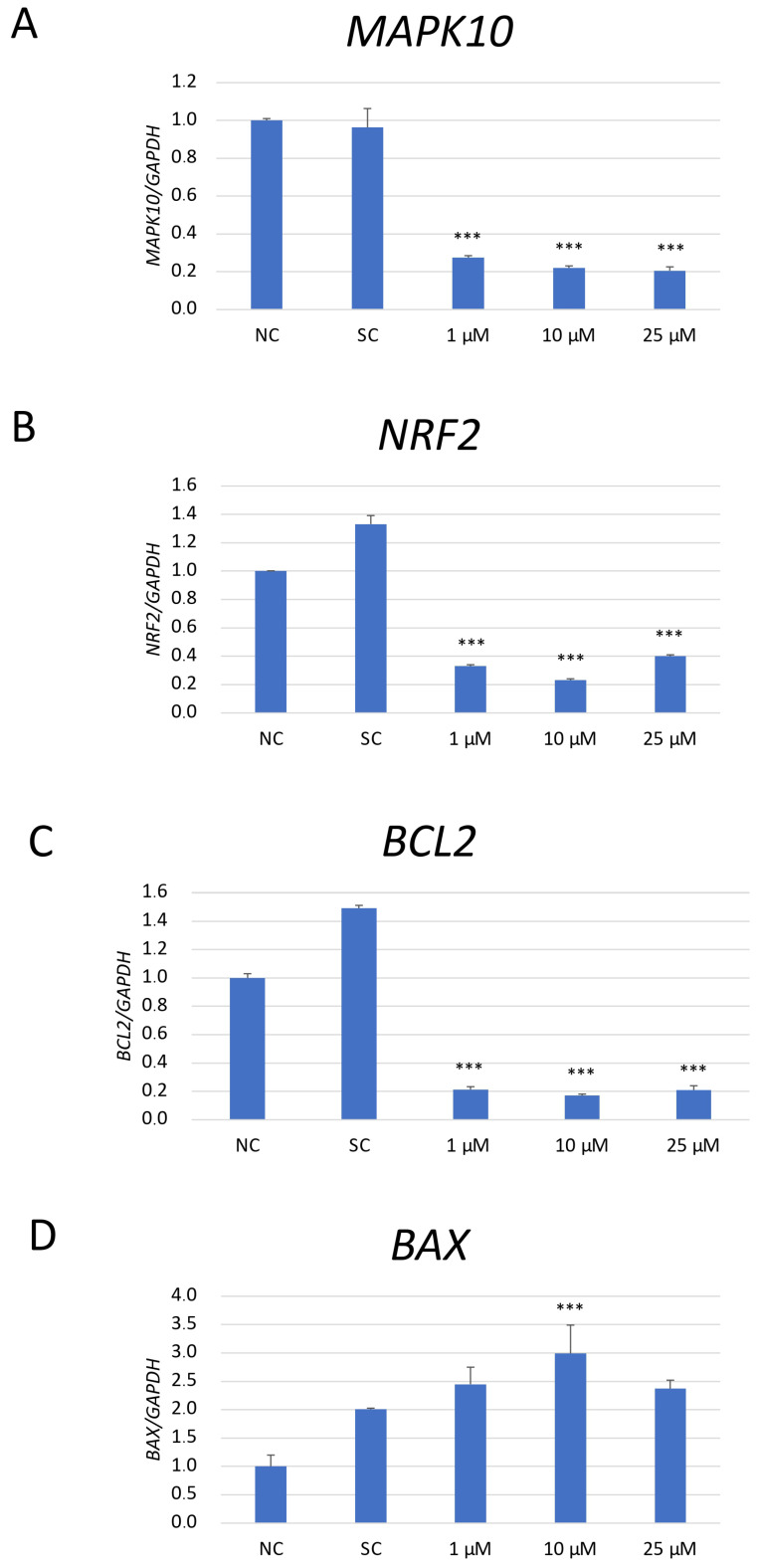
The mRNA depression levels of MAPK10 (**A**), NRF2 (**B**), BCL2 (**C**), and BAX (**D**) genes in SH-SY5Y cells treated with JNK at 1 μM, 10 μM, and 25 μM for 24 h. GAPDH was used as a reference gene. NC—negative control (untreated cells); SC—solvent control (cells treated with 0.01% DMSO). All experiments were run in triplicate. Statistics: One-way ANOVA with Bonferroni post hoc test (mean ± SD); *** *p* < 0.001 vs. negative control.

The expression analysis of mRNA of genes associated with apoptosis (Mitogen-activated protein kinase 10/c-Jun N-terminal kinase 3 (MAPK10/JNK3), B-cell lymphoma 2 (BCL2), and BCL2-Associated X, Apoptosis Regulator (BAX)) and anti-oxidative response (nuclear factor erythroid 2-related factor 2 (NRF2)) was performed in SH-SY5Y cells exposed to JNK V (1 μM, 10 μM, 100 μM), 0.01% DMSO (solvent control), and untreated cells (negative control) (Figure 6). As NRF2 is a master regulator of antioxidant responses, and JNK can modulate oxidative stress response signalling, the changes in NRF2 mRNA expression could explain whether the observed anti-neoplastic effects could be related to oxidative stress regulation rather than only direct apoptotic signalling [33]. The obtained results have shown that the expression of MAPK10 mRNA, the less explored JNK isoform with an established neuroprotective effect on normal cells, was significantly reduced by all tested concentrations of JNK V compared to that in the negative control cells. JNK V in all tested concentrations caused a significant decline in the expression of anti-apoptotic BCL2 and a significant increase in the expression of pro-apoptotic BAX mRNA in comparison to that in the negative control. The expression of the master regulator of anti-oxidative responses, NRF2 mRNA, was significantly reduced by all tested concentrations of JNK V, indicating that JNK inhibition may impair SH-SY5Y resistance to oxidative stress by downregulating Nrf2-dependent protective mechanisms. These results suggest that JNK V modulates the expression of the chosen genes associated with apoptosis and oxidative stress response in SH-SY5Y cells, enhancing NB cell apoptosis.

### 2.7. Evaluation of Apoptosis- and Cell Cycle-Related Protein Expression in SH-SY5Y Cells After JNK Pharmacological Inhibition

**Figure 7:**
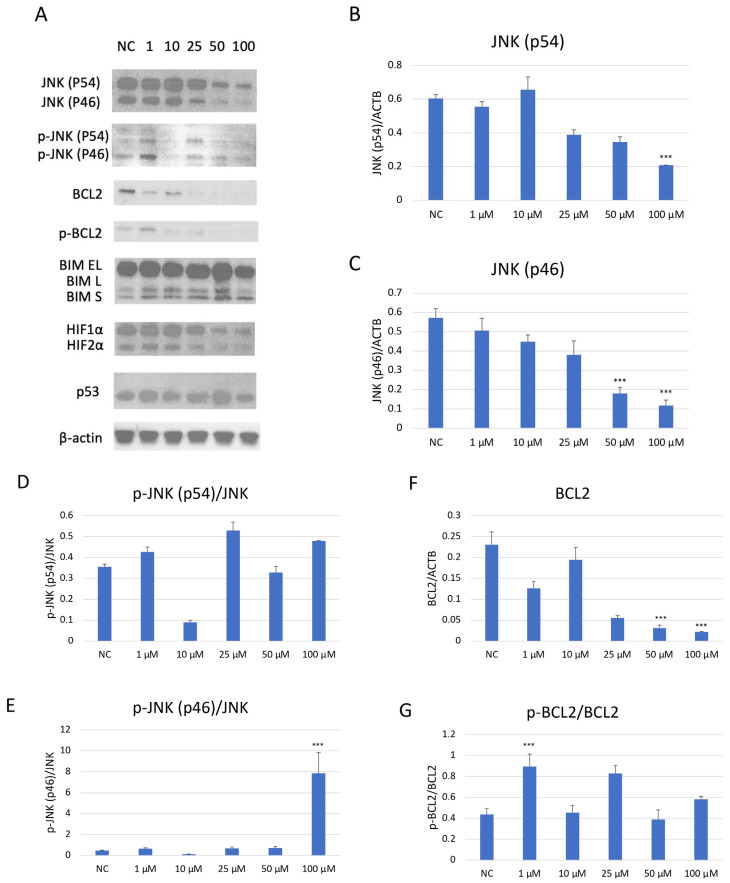

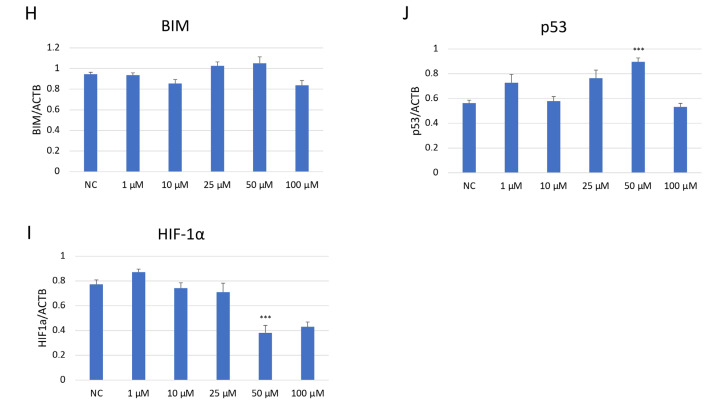
Western blot analysis (**A**) of the expression level of proteins JNK (p54) (**B**), JNK (p46) (**C**), p-JNK (p54) (**D**), p-JNK (p46) (**E**), BCL2 (**F**), p-BCL2 (**G**), BIM (**H**), HIF-1α (**I**), and p53 (**J**) in SH-SY5Y cells treated with JNK V at concentrations 1–100 μM for 24 h. NC—negative control; 1, 10, 25, 50, 100—increasing concentrations of JNK V in µM; ACTB (β-actin) was used as a loading control. All experiments were run in triplicate. Statistics: One-way ANOVA with Dunnett’s post hoc test (mean ± SD). *** *p* < 0.001 vs. negative control.

Western blot analysis demonstrated a dose-dependent suppression of JNK pathway activation in SH-SY5Y cells after JNK V treatment (Figure 7). The investigated compound treatment decreased both JNK isoforms p54 and p46 and modulated p-JNK isoform expression in a dose-dependent manner. Moreover, the deregulation of chosen key apoptosis- and cell cycle-related protein expression in SH-SY5Y cells by JNK V was demonstrated. The expression of anti-apoptotic Bcl-2 was significantly downregulated by JNK V at 50 and 100 µM, accompanied by a reduction in p-Bcl2 expression. The Bim expression levels after JNK V treatment were not changed significantly, but an increasing tendency of protein expression with higher concentrations of JNK V could be observed, which is consistent with a pro-apoptotic shift, since the Bcl-2 level is decreasing. The expression level of HIF-1α was reduced by all tested concentrations of JNK V, with statistical significance at 50 µM, suggesting a probable effect of JNK V on hypoxia-related signalling or stability. The p53 protein expression level was significantly increased after JNK V treatment at 50 µM, suggesting that the pro-survival p53 axis is affected by JNK inhibition under these conditions. The obtained results show that JNK signalling in SH-SY5Y cells can be pharmacologically blocked by JNK V to effectively suppress its downstream phosphorylation events and to promote an apoptotic molecular profile with reduced Bcl-2 signalling and increased p53 expression.

### 2.8. Evaluation of the Mitochondrial Metabolism of SH-SY5Y Cells After JNK Inhibition

**Figure 8:**
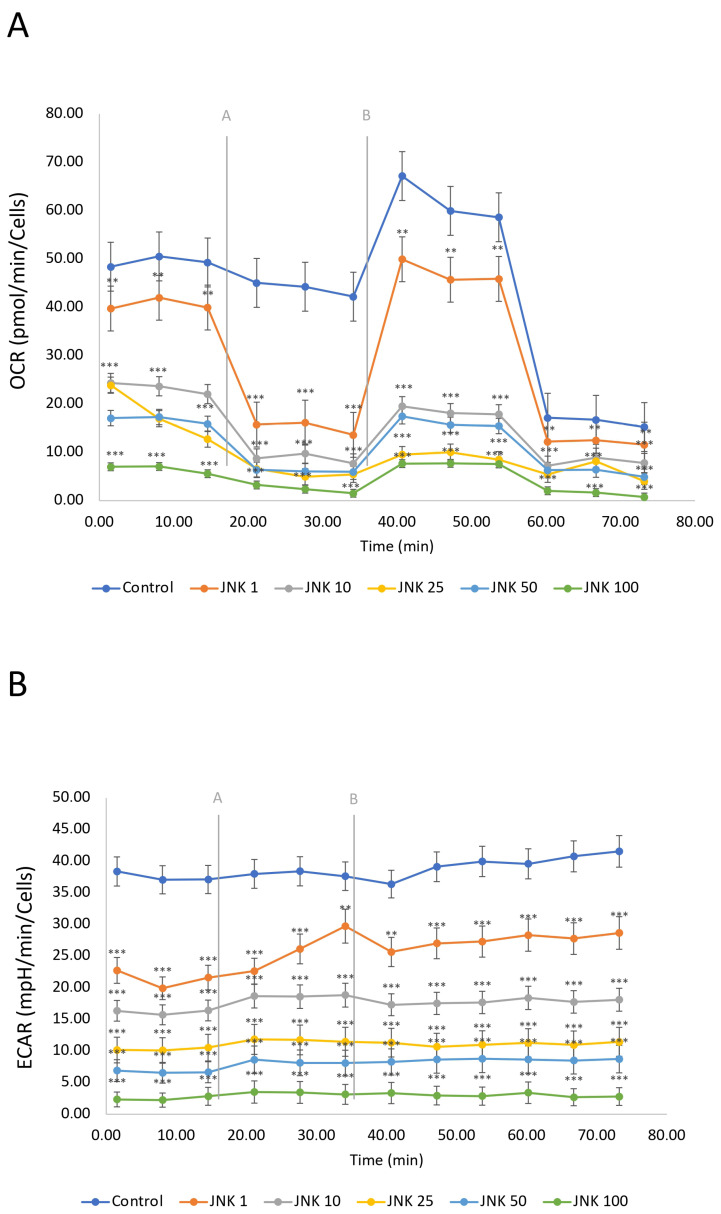
A real-time evaluation of mitochondrial bioenergetics—oxidative phosphorylation (**A**) and glycolysis (**B**) by Seahorse ATP Rate assay in SH-SY5Y cells treated with the JNK V inhibitor at 1 µM, 10 µM, 25 µM, 50 µM, and 100 µM. Control—negative control (untreated cells); grey letters and lines represent the injections of oligomycin [A] and rotenone/antimycin A [B]. All experiments were run in triplicate. Statistics: Student’s *t*-test (mean ± SD). *** *p* < 0.001, ** *p* < 0.01 vs. negative control.

The mitochondrial respiration, including oxidative phosphorylation, defined as oxygen consumption rate (OCR), and glycolysis, defined as extracellular acidification rate (ECAR), was measured using the Seahorse Mitostress Assay (Figure 8). SH-SY5Y cells were treated with JNK V inhibitor (1–100 µM) for 24 h. The OCR of untreated cells (control) was the highest, indicating active mitochondrial respiration of the living cells. The modulation of OCR in untreated cells is a consequence of injections of the assay reagents. The ECAR in the control group was relatively high and stable, indicating active glycolysis. JNK V treatment showed a significant reduction in OCR in a concentration-dependent manner compared to the control, suggesting that JNK inhibition decreases mitochondrial respiration in a dose-dependent manner. Similarly to the OCR results, ECAR decreased with increasing JNK inhibitor concentrations. The drop in both OCR and ECAR suggests that JNK inhibition not only impairs ATP production via mitochondria but also limits the cells’ ability to compensate through glycolysis, ultimately leading to a significant metabolic disruption, preserving only residual OCR while treated with 100 μM JNK V. These findings suggest that JNK activity may be critical for maintaining both oxidative phosphorylation and glycolysis in SH-SY5Y cells.

## 3. Discussion

The treatment of NB remains challenging because of its high genetic, immunological, and clinical heterogeneity; suboptimal response to initial therapy; high risk of relapse; and growing chemoresistance of tumour cells [6,7,8]. Therefore, the development of novel therapies should focus on increasing response rates to first-line therapies (chemo/immunotherapy), developing effective salvage therapies for relapsed and refractory disease, sustaining disease remissions, and enhancing antitumour immune function [34]. In infants, NB often regresses spontaneously or matures into ganglioneuroblastoma (GNB) after initial chemotherapy. However, the prognosis of NB patients becomes worse as patients age, and the current treatment standards remain ineffective in older patients and those with disseminated or recurrent disease. Despite advances in therapy, outcomes for high-risk patients remain poor [35,36].

*MYCN* oncogene amplification is the first independent prognostic factor indicating poor clinical outcomes of NB patients, observed in approximately 18% of cases, accounting for 40% of high-risk neuroblastomas [5,37]. The *MYCN* gene consistently promotes the progression of *MYCN*-related neuroendocrine tumours by driving dynamic spatial and temporal interactions, including distinct transcriptional programmes, DNA damage repair, resolving torsional stress, regulating R-loops, metabolic networks, stress–response, and apoptosis-related signalling patterns, and contributing to the aggressive phenotype of *MYCN*-amplified diseases [38,39]. However, despite the extensive study of *MYCN*-amplified NB, a gap in pre-clinical and clinical research of *MYCN* non-amplified cases was recently highlighted. New large-scale transcriptional subtyping analysis has suggested that *MYCN* non-amplified NBs are roughly heterogeneous and could be classified into three subgroups according to their transcriptional profiling [3]. Subgroup 2 had the worst prognosis, presenting an ‘*MYCN*’ signature that was potentially affected by overexpression of Aurora Kinase A (AURKA), while subgroup 3 showed an ‘inflamed’ immune-related gene signature. These findings emphasised the substantial extent of inner heterogeneity within the *MYCN* non-amplified population and show the vulnerability of stratified subgroups to different therapeutic approaches. Moreover, this research demonstrated active stress–response pathways and MAPK/JNK signalling within the subtype groups, underscoring that JNK pathway biology is not limited only to *MYCN*-amplified NB phenotypes. Consistent with this, JNK activity has been implicated in migration, invasion, and stress-induced apoptosis in both *MYCN*-amplified and *MYCN*-non-amplified neuroblastoma models [20,40]. Thus, although our study focuses on a non-amplified cell line, it addresses cellular mechanisms relevant to multiple NB subtypes.

Furthermore, NB with *MYCN* amplification exhibits both transcriptional and functional inhibition of JNK signalling, resulting in low basal JNK activity and a significantly impaired apoptotic response. Patient-specific modelling consistently places tumours with *MYCN* amplification in the low JNK activity cluster, and system-level reconstructions show systematic downregulation of upstream JNK circuits in *MYCN*-induced disease [41,42]. Notably, a newly published study has shown that high-risk tumours with *MYCN* amplification respond better to treatment regimens involving JNK-independent drugs, confirming that the JNK pathways are significantly silenced in this subtype [43]. Hence, JNK-dependent therapies are much more biologically relevant in *MYCN*-nonamplified neuroblastoma, where the JNK cascade remains intact.

Elevated JNK activity is an established contributor to malignancy progression and chemo/radiotherapy resistance in many cancers in which JNK is transiently activated, such as B-cell lymphoma, osteosarcoma, breast, lung, skin, and pancreatic cancer [13,27,44,45,46]. JNK exhibits pro-survival function in cancer cells due to its ability to enhance the processes of proliferation, migration, and invasion through a plethora of mechanisms, including synergistic action with p38 MAPKs and nuclear factor kappa B (NF-κB), the upregulation of antiapoptotic gene expression such as BCL2, and the blockade of caspase activation [23,47,48,49]. Moreover, JNK induces pro-survival autophagy to counteract apoptosis, which, together with immune evasion mechanisms, increases the resistance of cancer cells to chemotherapy [50,51]. Therefore, JNK constitutes an interesting molecular anti-neoplastic target for therapeutic intervention with small-molecule kinase inhibitors [52]. Intriguingly, the JNK inhibitor AS602801, bentamapimod, was evidenced to block gap-junction communication between astrocytes and glioma cells [53], lung cancer stem cells [54], and breast cancer cells, and together with modulating Cx43 expression, it inhibited particular cancer metastasis to the brain [24]. Multiple JNK targeting strategies, including various ATP-competitive, non-kinase, and substrate-competitive inhibitors, have been developed and, due to the anticancer potential demonstrated in preclinical models, several JNK inhibitors have been investigated in the II/III phases of clinical trials [55,56]. However, JNK’s pleiotropic signalling limits the clinical application of direct JNK inhibition, as evidenced by past clinical trials primarily focused on fibrotic and inflammatory diseases [40,57,58,59,60].

The anticancer effects of the inhibition of the JNK signalling pathway have previously been successfully reported in diverse NB in vitro and in vivo models [61]. The newest data indicated an enormous potential of JNK inhibitors in NB treatment, as they have been reported to induce NB cell death via direct activation of p53, Bcl-2, and caspase-dependent pathways [27,62]. NSC697923, an inhibitor of the ubiquitin-conjugating enzyme E2 N, promoted NB cell death by regulating both p53 and JNK pathways, apoptosis induction, and colony formation inhibition in a dose-dependent manner across multiple MYCN-amplified and MYCN-non-amplified NB cell lines [29]. The compound also demonstrated in vivo antineoplastic activity in NB orthotopic xenografts. Bortezomib, a proteasome inhibitor, suppressed cell growth and angiogenesis in SH-SY5Y and CHP126 NB cell lines by modulating the JNK pathway, while its combination with all-trans retinoic acid enhanced tumour growth inhibition in human NB xenografts and a mouse model [63,64,65]. Recently, a highly selective in vitro JNK3 Inhibitor, FMU200, was reported to decrease cell viability in SH-SY5Y cells [66,67]. The newest data showed that another selective JNK3 inhibitor, piceatannol, was highly effective in apoptosis induction by the inhibition of Cyt-C/ Bcl-2/caspase-3-dependent pathway, protecting SH-SY5Y cells from hypoxic insult [68,69]. The other possible mechanism of activity of JNK inhibitors in NB is a disruption in cancer stemness maintained by the STAT3-JNK axis, evidenced by the remarkably lower viability of IMR5, NLF, and SK-N-AS NB cell lines, and the downregulation of the expression of the stem cell marker CXCR4 after treatment with SP600125 or JNK-IN-8 inhibitors [70,71].

Our study is the first to comprehensively investigate the anti-neoplastic effect of AS601245 (JNK inhibitor V) on the SH-SY5Y cell line via a detailed assessment of the cellular effects of the compound on cell functions. JNK V demonstrated a dose-dependent reduction in the proliferation of T-cell acute lymphoblastic leukaemia (T-ALL) cells by inducing apoptosis and cell cycle arrest, accompanied by a reduction in c-Myc and Bcl-2 protein levels [72]. Moreover, JNK V significantly diminished the adhesion and migration of multiple human colon cancer cell lines through specific gene expression modulation [73,74,75]. Recent gene analysis has reported that the JNK V inhibitor also shows promise for use in breast cancer treatment [13,24]. However, the potential of JNK V has not yet been elucidated in any pre-clinical or clinical trials with regard to NB. Importantly, the JNK V inhibitor has the most potent impact on the least-explored isoform of JNKs, JNK3. As NB is a neural crest-derived malignancy with a high propensity for metastasis, we selected this compound for its high specificity; strong pro-apoptotic effects, evidenced in other cancers; and its neuroprotective and anti-inflammatory properties, which may help minimise excessive toxicity to neural tissue [76,77,78,79].

An important notion that emerges from our study is that JNK inhibition induced by the chosen inhibitor is not cytotoxic towards primary human Schwann cells (HSC)—a well-established control model for NB in vitro research. Furthermore, JNK inhibition may also modulate the tumour microenvironment (TME) by influencing stromal development, specifically Schwannian stroma, which plays a crucial role in NB to GNB maturation [80,81]. Our results demonstrated that JNK inhibitor treatment reduced the invasion abilities of SH-SY5Y cells, so it is reasonable to hypothesise that JNK inhibition could favour the development of a more organised, Schwannian-rich stroma. JNK inhibitors might induce a more permissive environment for stromal differentiation and maturation by reducing tumour cell invasiveness, which may shift the NB cells towards a phenotype closer to GNB, which is associated with better prognosis [81,82]. Also of particular value in our research is the Seahorse mitochondrial metabolism analysis, which reveals a novel aspect of JNK V’s anti-neoplastic mechanism—significant disruption of both oxidative phosphorylation and glycolysis—which, together with pro-apoptotic gene regulation, ultimately contributes to its overall antineoplastic efficacy.

A major limitation of our in vitro study is that all experimental data are generated in a single neuroblastoma cell line, SH-SY5Y, which is an *MYCN*-non-amplified NB cell model that represents only low- and intermediate-risk NB. To investigate the effect of JNK inhibition on an appropriate model of high-risk NB patients and to be able to ensure the generalizability of the results, further experiments on *MYCN*-amplified cell lines are mandatory. Furthermore, future research should include not only additional NB cell lines but also primary tumour-derived cells, supportive stromal cell types, and in vivo studies to better understand tumour cell death mechanisms and more accurately reflect the TME and disease complexity. Secondly, exclusive pharmacological JNK inhibition with only a single ATP-competitive inhibitor was tested. Further genetic validation, including JNK silencing, knockout models, or rescue strategies, is necessary to confirm the mechanistic basis of the observed effects. As an exclusive reliance on a single inhibitor is limited, additional orthogonal approaches would further strengthen causal inference and also exclude off-target effects. Thirdly, another important note is that the NB cell death observed in our cytotoxicity experiments may not be solely attributed to apoptosis, but also to alternative mechanisms such as necrosis or necroptosis, which, though not examined in this study, could also have contributed to the observed effects. Fourthly, and moreover, the short 24 h incubation period in toxicity and mechanistic experiments may capture only the acute, short-term effects and cellular responses of JNK inhibition in NB cells, necessitating extended exposure times to access long-term cellular responses and long-term toxicity. Further experiments with longer incubation times are necessary.

## 4. Materials and Methods

### 4.1. Cell Culture

The SH-SY5Y human neuroblastoma cell line (ATCC, Manassas, VA, USA) is a thrice-cloned subline of the SK-N-SH cell line and constitutes a well-established, neuronally relevant NB in vitro model [83,84]. The cells were cultured in Eagle’s minimum essential medium (EMEM) (ATCC, Manassas, VA, USA) supplemented with 10% heat-inactivated foetal bovine serum (FBS) (ATCC, Manassas, VA, USA) as well as 1% penicillin–streptomycin (ScienCell, Carlsbad, CA, USA). As a relevant control for NB in vitro research, primary Human Schwann Cells (HSC) (ScienCell, Carlsbad, CA, USA) isolated from the human spinal nerve were used. These neural crest-derived cells ensheathe and myelinate axons of peripheral nerves, and interact with cancerous cells in the tumour microenvironment (TME) [85]. The cells were cultured in Schwann Cell Medium (ScienCell, Carlsbad, CA, USA). As an additional control, widely used in toxicology and cellular biology research, the BJ cell line (ATCC, Manassas, VA, USA)—human fibroblasts from normal foreskin—was used [86,87]. Cells were cultured in EMEM supplemented with 10% FBS, 200 mM L-glutamine (Sigma-Aldrich, Saint Louis, MO, USA), and 1% antibiotics solution. Using those two different normal cell lines as controls allows us to evaluate both the relevance and broader safety profile of the tested compound. All cell lines were cultured in the same incubator at standard conditions (37 °C, 5% CO_2_, 95% humidity). Every 2 to 3 days, the cell culture media were replaced. After the cells reached 70–80% confluence, cell passages were performed, with a brief rinse with Dulbecco’s Phosphate-Buffered Saline (DPBS) (ScienCell, Carlsbad, CA, USA), and detachment using 0.25% Trypsin-EDTA solution (ScienCell, Carlsbad, CA, USA). For each experiment, the cell culture was not expanded beyond passage 15.

### 4.2. Inhibitor Treatment

To selectively inhibit the JNK pathway, we have used the commercially available JNK inhibitor AS601245 (JNK inhibitor V, JNK V)—1,3-Benzothiazol-2-yl-(2-{[2-(3-pyridinyl)ethyl]amino}-4-pyrimidinyl) acetonitrile (Sigma-Aldrich, Saint Louis, MO, USA). It is a potent, reversible, and cell-permeable ATP-competitive inhibitor of c-Jun N-terminal kinases (IC_50_ values: 150 nM for hJNK1, 220 nM for hJNK2, and 70 nM for hJNK3) with anti-inflammatory characteristics and a 10- to 100-fold better selectivity compared to a panel of 25 other commonly researched kinases [88,89]. The JNK V showed notable antineoplastic properties in multiple T-ALL and colon cancer in vitro studies [27,74,75]. Importantly, it exerts the strongest inhibitory effect on the JNK3 isoform, whose role in tumours is not fully established. The inhibitor’s efficacy and well-established in vivo safety profile have been demonstrated in gerbils, mice, and rats following oral, intraperitoneal, and intravenous administration [88,90]. The compound was reconstituted in dimethyl sulfoxide (DMSO) (Sigma-Aldrich, Saint Louis, MO, USA) and maintained at −20 °C in darkness. The vehicle concentration in the culture medium was not more than 0.01%. Furthermore, cultures treated with 0.01% DMSO alone were used to rule out the potential effects of the vehicle.

### 4.3. Cytotoxicity Measurement

The cytotoxicity assessment of the evaluated JNK V compound was conducted using the 2,3-bis-(2-methoxy-4-nitro-5-sulfophenyl)-2H-tetrazolium-5-carboxanilide (XTT) colorimetric assay (Thermo Scientific, Waltham, MA, USA). The experiment was performed on SH-SY5Y, HSC, and BJ cell lines. Cells were seeded at a density of 5 × 10^3^ cells/well in a 96-well plate and cultured in 100 μL of the complete growth medium for 24 h. The cells were then treated and incubated with a particular compound for a standardised time of 24 h to assess the short-term effects of the investigated compound. SH-SY5Y cells were exposed to a wide concentration range of JNK V (0.1–100 µM). HSC and BJ cells were treated with JNK V at concentrations of 0.1–25 µM, because of their substantial sensitivity to DMSO at the concentration required to dissolve higher doses of the investigated compound, specifically 50 and 100 µM JNK V, together with the reduced solubility and partial precipitation of the compound, specifically in Schwann Cell Medium. Cells treated with 20% DMSO served as a positive control. Cells treated with 0.01% DMSO served as the solvent control, while untreated cells constituted the negative control. After incubation for 24 h, 75 μL of XTT/PMS suspension was added to each well in accordance with the manufacturer’s protocol. After 2 h of incubation in a 5% CO_2_ incubator at 37 °C, the absorbance measurement was performed using the Synergy HT spectrophotometer (BioTek, Shoreline, WA, USA) at a wavelength of 450 nm.

### 4.4. Morphology Assessment

The morphology of SH-SY5Y cells was assessed using an inverted microscope (Nikon, Tokyo, Japan), after 24 h incubation of the cells with, respectively, 0.1–100 µM JNK V in the experimental group, 0.01% DMSO in the solvent control group, or 20% DMSO in the positive control group. For each experimental condition, at least 10 random fields were acquired, capturing around 200 cells per replicate. Images were captured at 10× magnification, under identical exposure settings. All images were saved in TIFF format. Morphological changes, including cell shape, adhesion, neurite outgrowth, cytoplasmic integrity, and nuclear condensation, were analysed qualitatively.

### 4.5. Colony-Forming Assessment

A clonogenic assay, which assesses the ability of a single cell to form a colony over 14 days, was performed on SH-SY5Y cells to evaluate their proliferation and survival abilities. Cells treated with 0.1–25 μM JNK V constituted the experimental control, while untreated cells served as a negative control [91]. Firstly, cells at a density of 2 × 10^3^ cells/well were seeded on a 12-well plate with EMEM supplemented with 10% FBS. After being incubated for 72 h, the JNK V inhibitor was applied. The media was refreshed every 3 days, and cells were grown for a total of 2 weeks. Ultimately, the cells were washed with DPBS, fixed in 10% neutral buffered formalin (NBF) (Sigma-Aldrich, Saint Louis, MO, USA) for 30 min, and visualised using 0.1% crystal violet (Sigma-Aldrich, Saint Louis, MO, USA) for 30 min. The plate was then allowed to dry. The colony-forming abilities of cells were assessed using an inverted microscope (Nikon, Tokyo, Japan). For each experimental condition, at least 10 random fields were acquired, capturing around 50 cells per replicate. Images were captured at 400× magnification, under identical exposure settings. Confluence was quantified based on the percentage of the cell-covered surface area within randomly selected 100 × 100 µm image fragments. The proportion of the area covered by cells was calculated using ImageJ software (version 1.53, National Institutes of Health, Bethesda, MD, USA).

### 4.6. Migration Assessment

The wound healing (scratch) assay was performed to assess the migration abilities of SH-SY5Y cells after treatment with JNK at concentrations of 0.1–3 μM, which maintained cell viability over 80%, confirmed by a previous XTT assay [92]. Cells were seeded in a 24-well plate at a density of 2 × 10^5^ cells/mL in an EMEM with 10% FBS. After reaching full confluence, a 200 μL sterile plastic tip was used to scrape the cell monolayer longitudinally on each well. All wells were then intensively rinsed three times with DPBS to mechanically eliminate non-adherent floating cells. To suppress the contribution of cell proliferation but allow cells to survive for the following 72 h, cells were incubated in a medium supplemented with a low serum concentration (1% FBS) together with an inhibitor at an appropriate concentration. Untreated cells constituted a negative control. Subsequently, the scratched area was carefully viewed, and representative images were captured at 0 h, 24 h, 48 h, and 72 h after wounding time points using an inverted phase-contrast microscope (Nikon, Tokyo, Japan). Images were captured at 100× magnification, under identical exposure settings. All images were saved in TIFF format and analysed using ImageJ software (version 1.53, National Institutes of Health, Bethesda, MD, USA). The percentage of wound closure was calculated by identifying the low-texture region corresponding to the acellular wound area in the Laplacian texture profile and expressing the reduction in this area relative to the initial wound width, as recommended [93].

### 4.7. Apoptosis Evaluation

The Caspase-3 colorimetric activity assay (Abcam, Cambridge, UK) was performed to assess the effect of JNK inhibition on the apoptosis of SH-SY5Y cells. Cells treated with 0.75–100 µM of JNK V for 24 h constituted the experimental sample, the negative control comprised untreated cells, whereas positive control cells were treated with 10 μM staurosporine for 24 h. Following incubation, the cells were dissociated with Trypsin/EDTA solution and centrifuged. Subsequently, the samples were prepared for further protein isolation by resuspension of the cell pellet in 50 μL of cold lysis buffer, incubation for 10 min on ice, centrifugation at 10,000× *g* for 1 min, transfer of supernatant to new microcentrifuge tubes, and maintenance on ice. Then, the Pierce^TM^ Bicinchoninic Acid (BCA) Protein Assay (Thermo Scientific, Waltham, MA, USA), calibrated to 100 μg of protein per sample, was performed to determine the protein concentration. Briefly, each well of the microplate was filled with 50 μL of sample per well (except for background wells), 50 μL of freshly prepared Caspase Reaction Mix using 2X Reaction Buffer with dithiothreitol (DTT), 5 μL of the substrate solution (4 mM Asp-Glu-Val-Asp-para-nitroanilide (DEVD-pNA)), and then incubated for 120 min at 37 °C. By monitoring the samples’ absorbance at 400 nm in the Synergy HT Microplate Reader (BioTek Instruments, Winooski, VT, USA), the quantity of p-NA was measured quantitatively.

### 4.8. Gene Expression Analysis

The expression of selected genes connected with apoptosis and cellular damage control was evaluated by qRT-PCR analysis in cells treated with JNK V at 1 μM, 10 μM, and 25 μM and 0.01% DMSO as a solvent control. Untreated cells constituted the control. Following incubation with the investigated compound, the PureLinkTM RNA Mini Kit (Invitrogen, Waltham, MA, USA) was used to extract the total RNA, in line with the manufacturer’s guidelines. Then, the obtained samples’ RNA levels were measured and normalised by use of the Synergy HT Microplate Reader (BioTek Instruments, Winooski, VT, USA). Subsequently, using the High-Capacity cDNA Reverse Transcription Kit (Applied Biosystems, Waltham, MA, USA), a final concentration of 100 ng of cDNA was produced by transcription of the isolated RNA, according to the manufacturer’s protocol. Then, the expression profile of the MAPK10 (Hs00959268_m1), BCL2 (Hs00608023_m1), BAX (Hs00180269_m1), and NRF2 (Hs00232352_m1) was analysed using the TaqMan^TM^ Gene Expression Assays (Applied Biosystems, Waltham, MA, USA). GAPDH (Hs99999905_m1) served as a reference gene. The overall reaction volume was 20 μL, containing 1 μL probes, 1 μL cDNA, 10 μL TaqMan^TM^ Universal PCR Master Mix II (Applied Biosystems, Waltham, MA, USA), and 8 μL nuclease-free water (Invitrogen, Waltham, MA, USA). Ultimately, the Bio-Rad CFX96 system (Bio-Rad, Hercules, CA, USA) was used to conduct the PCR reaction in the following sequence: initial denaturation (15 min, 95 °C); cycling–denaturation (10 s, 95 °C); and annealing/extension (60 s, 60 °C), for 40 cycles each. All collected data was quantified using 2^−∆∆Ct^ values.

### 4.9. Protein Expression Analysis

The effect of JNK V on the expression of several key apoptosis- and cell cycle-related proteins in SH-SY5Y cells was evaluated by Western blot analysis. The JNK V inhibitor was applied to the cells for 24 h at doses of 1, 10, 25, 50, and 100 µM. The untreated cells constituted the negative control. Following treatment, the cells were collected from the wells, and the MinuteTM Total Protein Extraction Kit (Invent Biotechnologies, Plymouth, MN, USA) was used to extract the total protein. The PierceTM BCA Protein Assay (Thermo Scientific, Waltham, MA, USA) was used to assess and normalise the protein quantities in the samples. The protein samples were denatured at 70 °C for 10 min, placed into gel wells, and subsequently electrophoresed for 50 min using the NuPageTM/XCell SureLockTM system (Invitrogen, Waltham, MA, USA). After that, the proteins were moved to a PVDF membrane (Invitrogen, Waltham, MA, USA) and incubated for one hour. The membrane was then blocked for one hour in a solution of 5% BSA for phosphoproteins, or skim milk for non-phosphoproteins (BioShop Canada, Burlington, ON, Canada) in 1X TBST (Thermo Scientific Chemicals, Waltham, MA, USA). Then, the membranes were incubated for 24 h with primary monoclonal antibodies for targeted proteins such as JNK, p-JNK, Bim, Bcl-2, p-Bcl-2, p53, and HIF-1α (dilution 1:1000; Cell Signaling Technology, Danvers, MA, USA). The antibodies’ ID numbers are listed in Table 1. The loading control was β-actin. The next day, secondary HRP-linked antibodies (dilution 1:5000; Cell Signaling Technology, Danvers, MA, USA) were added to the membranes after they had been cleaned three times with 1X TBST. Following a final wash in TBST, the membrane was exposed to SuperSignal^TM^ West Pico Chemiluminescent Substrate (Thermo Scientific, Waltham, MA, USA) for five minutes in the dark. The ChemiDoc^TM^ Imaging System (Bio-Rad, Hercules, CA, USA) was used to detect the protein bands using enhanced chemiluminescence. NIS-Elements Advanced Research software version 5.42 (Nikon, Tokyo, Japan) was used to conduct the densitometry analysis.

### 4.10. Cellular Bioenergetics Assessment

The Agilent Seahorse XFp Real-Time ATP rate assay (Agilent Technologies, Santa Clara, CA, USA) was performed to evaluate the effect of JNK inhibition on SH-SY5Y cells’ cellular respiration. After being seeded in Seahorse XF HS Miniplates (Agilent Technologies, Santa Clara, CA, USA), the cells were exposed to 1, 10, 25, 50, and 100 μM of JNK V. Untreated cells constituted the control. The day before the experiment, the Seahorse XFp Sensor Cartridge was hydrated overnight in a non-CO_2_ incubator as directed by the manufacturer. The cartridge was rehydrated using Seahorse XF Calibrant (Agilent Technologies, Santa Clara, CA, USA) and incubated for 45 min on the day of the test. Following the manufacturer’s instructions, the culture medium was removed from each well, and the cells were washed with preheated assay medium (pH 7.4) that contained Seahorse XF DMEM, 10 mM glucose, 2 mM L-glutamine (Agilent Technologies, Santa Clara, CA, USA), and 1 mM sodium pyruvate. Cells were cultured at 37 °C in a non-CO_2_ incubator for 45 min to attain the desired temperature and pH before measurement. Then, the ATP synthase inhibitor oligomycin (2.5μM) and the mitochondrial complex I/III inhibitors rotenone/antimycin A (0.5 μM) solutions were inserted into suitable cartridge ports, in line with the manufacturer’s instructions. After the initial three baseline measurements using a Seahorse XF HS Mini Analyser (Agilent Technologies, Santa Clara, CA, USA), three measurements were taken after each injection of chemical compounds.

### 4.11. Statistical Analysis

The statistical analysis was performed using Statistica (version 13; StatSoft, Kraków, Poland). The normality of data distribution was determined by the Shapiro–Wilk test. If data were homogeneous and normally distributed, one-way ANOVA with Bonferroni or Tukey’s post hoc testing was performed for comparison between multiple groups. Otherwise, the Kruskal–Wallis one-way analysis of variance on ranks with Dunn’s post hoc test with Bonferroni correction was performed. All studies were performed in triplicate, and the data are presented as mean ± SD. The following symbols indicate statistically significant differences in the graphs: * *p* < 0.05, ** *p* < 0.01, *** *p* < 0.001.

## 5. Conclusions

To our knowledge, this is the first comprehensive study to characterise the acute in vitro effects of JNK inhibitor AS601245 (JNK V) on the human *MYCN*-non-amplified neuroblastoma cell line SH-SY5Y. We demonstrate that JNK V reduces SH-SY5Y cell viability and dysregulates key cellular functions, without exerting cytotoxicity toward normal human Schwann cells and fibroblasts under the conditions tested. JNK V induces a dose-dependent inhibition of proliferation, colony formation, and migration, accompanied by increased caspase-3 activity, pro-apoptotic gene and protein expression, and significant disruption of both oxidative phosphorylation and glycolysis. These results provide a detailed description of early cellular responses to pharmacological JNK inhibition in the *MYCN*-non-amplified neuroblastoma in vitro model.

## Figures and Tables

**Table 1:** The ID numbers of the applied primary antibodies (Cell Signaling Technology, Danvers, MA, USA).

Antibody Name	Catalogue Number
SAPK/JNK Antibody	#9252
Phospho-SAPK/JNK (Thr183/Tyr185) Antibody	#9251
Bcl-2 (D55G8) Rabbit mAb	#4223
Phospho-Bcl-2 (Ser70) (5H2) Rabbit	#2827
Bim(C34C5) Rabbit mAb	#2933
p53 Antibody	#9282
HIF-1α (D1S7W) XP^®^ Rabbit mAb	#36169
β-Actin (13E5) Rabbit mAb	#4970
Anti-rabbit IgG, HRP-linked Antibody	#7074

## Data Availability

The original contributions presented in this study are included in the article. Further inquiries can be directed to the corresponding author.

## Abbreviations

The following abbreviations are used in this manuscript:

BAX	BCL2-Associated X Apoptosis Regulator
BCL2	B-Cell Lymphoma 2
Cyt-C	Cytochrome C
DMEM	Dulbecco’s Modified Eagle Medium
DMSO	Dimethyl Sulfoxide
ECAR	Extracellular Acidification Rate
EMEM	Eagle’s Minimum Essential Medium
FBS	Foetal Bovine Serum
GNB	Ganglioneuroblastoma
HSC	Human Schwann Cells
JNK	C-Jun N-Terminal Kinases
MAPK	Mitogen-Activated Protein Kinase
MAPK10/JNK3	Mitogen-Activated Protein Kinase 10/C-Jun N-Terminal Kinase 3
NB	Neuroblastoma
NF-κB	Nuclear Factor Kappa B
NRF2	Nuclear Factor Erythroid 2-Related Factor 2
OCR	Oxygen Consumption Rate
TME	Tumour Microenvironment
T-ALL	T-cell Acute Lymphoblastic Leukaemia
